# Prophylactic AEDs Treatment for Patients With Supratentorial Meningioma Does Not Reduce the Rate of Perioperative Seizures: A Retrospective Single-Center Cohort Study

**DOI:** 10.3389/fonc.2020.568369

**Published:** 2020-12-04

**Authors:** Ming Yang, Yong-Ran Cheng, Meng-Yun Zhou, Ming-Wei Wang, Lan Ye, Zu-Cai Xu, Zhan-Hui Feng, Xun-Tai Ma

**Affiliations:** ^1^ Neurosurgical Department, Affiliated Hospital of Guizhou Medical University, Guiyang, China; ^2^ School of Public Health, Hangzhou Medical College, Hangzhou, China; ^3^ Department of Cardiology, Affiliated Hospital of Hangzhou Normal University, Hangzhou, China; ^4^ The Medical Function Laboratory of Experimental Teaching Center of Basic Medicine, Guizhou Medical University, Guiyang, China; ^5^ Neurological Department, Affiliated Hospital of Zunyi Medical University, Zunyi, China; ^6^ Neurological Department, Affiliated Hospital of Guizhou Medical University, Guiyang, China; ^7^ Neurological Department, The First Affiliated Hospital of Chengdu Medical College, Chengdu, China

**Keywords:** seizure, operation, prophylactic treatment, antiepileptic drugs, supratentorial meningioma, non-skull base location

## Abstract

Meningiomas, the most common brain tumor, inevitably require surgical treatment. However, the efficacy of prophylactic antiepileptic drugs (AEDs), in reducing the frequency of new-onset seizures during the perioperative period remains controversial. To further clarify if prophylactic antiepileptic drug treatment for patients with meningioma had value, we reviewed the medical records of 186 supratentorial meningioma patients who were operated at our hospital between 2016 and 2018. SPSS 24.0 software was used for statistical analysis. The results of univariate analysis showed that factors including age, sex, the course of the disease (years), maximum cross-sectional area of the tumor, location of the tumor, multiple or single tumors, adjacent to the cortex, peritumoral brain edema, World Health Organization classification, and peritumoral adhesion were not associated with perioperative seizures (*P* >0.05). Furthermore, the results of multivariate analysis revealed hydrocephalus (OR 4.87 P = 0.05) and non-skull base location (OR 1.88 P = 0.04) were significant risk factors for perioperative in-hospital seizures. Prophylactic valproic acid treatment did not contribute to the alleviation of perioperative seizures (OR 1.76 P = 0.04). However, Multivariate logistic regression analyses excluding the patients with seizures before operation confirmed prophylactic valproic acid treatment did not reduce the frequency of seizures during the perioperative period (OR 1.84 P = 0.04). Taken together, the data suggest that prophylactic valproic acid treatment for patients with supratentorial meningioma does not reduce the rate of perioperative seizures.

## Introduction

Meningiomas are very common tumors in the brain and account for approximately 36.4% of all brain tumors (1). Almost 90% of meningiomas are benign (1). The World Health Organization (WHO) classifies meningiomas as grades I, II, and III according to the characteristics of the histopathology (2). Computed tomography (CT) scan or magnetic resonance imaging (MRI) scan are usually used to confirm the initial diagnosis of meningioma (3). The incidence of meningioma increases with age (1).

Patients with meningiomas show a variety of different clinical symptoms, which is mainly due to the location of the meningioma in the brain (4, 5). Focal neurological deficits often occur because the meningioma affects the blood vessels or brain tissue (6). Seizures are a common symptom of meningiomas. Reports indicated that the incidence of preoperative epilepsy in meningiomas ranges from 15–39% (6–10).

The vast majority of patients receive surgical resection treatment (11); however, some patients have apparent seizures after surgery. Reily et al. (12) showed that the incidence of postoperative seizures was approximately 30% (12). In order to reduce the risk of seizures, some surgeons have utilized prophylactic AEDs (13). However, whether AEDs can help reduce perioperative seizures remains controversial.

Islim et al. published the review in 2017 (14). The data were collected from between January 1990 and November 2016. The conclusion showed the prophylactic valproic acid treatment could reduce the rate of perioperative seizures. They performed a retrospective cohort study in 2018 (15), and also got the same conclusion. Between 2017 and 2020, additional published articles were retrospective cohort studies (16–19). The results showed prophylactic AEDs treatment did not decrease the incidence of postoperative seizures of supratentorial meningioma. In order to further clarify if AEDs reduced the incidence of postoperative seizures, we collected the data of patients who underwent meningioma resections during the past 2 years in our hospital and performed a retrospective study.

## Methods

### Study Design

Our study was a retrospective study. All patients received surgical treatment in Affiliated hospital of Guizhou Medical University (Guizhou, China). Our project was approved by the Ethics Committee of Affiliated Hospital of Guizhou Medical University. Because our study was a retrospective cohort study, informed consent was not needed.

First, we collected all the risk factors that might affect the occurrence of postoperative seizures. Seizures during the perioperative period were recorded. We were primarily interested in the incidence of seizures during the perioperative period, when prophylactic AEDs are administered.

Second, we performed univariate analysis to observe which indicators might have an impact on the occurrence of perioperative seizures. Then, we used multivariate analysis to determine which factors might contribute to the occurrence of perioperative seizures.

Third, we excluded patients who had seizures before surgery. We then used univariate analysis and multivariate analysis to explore whether prophylactic AEDs decreased the incidence of perioperative seizures.

### Patients and Data Collection

**Figure 1:**
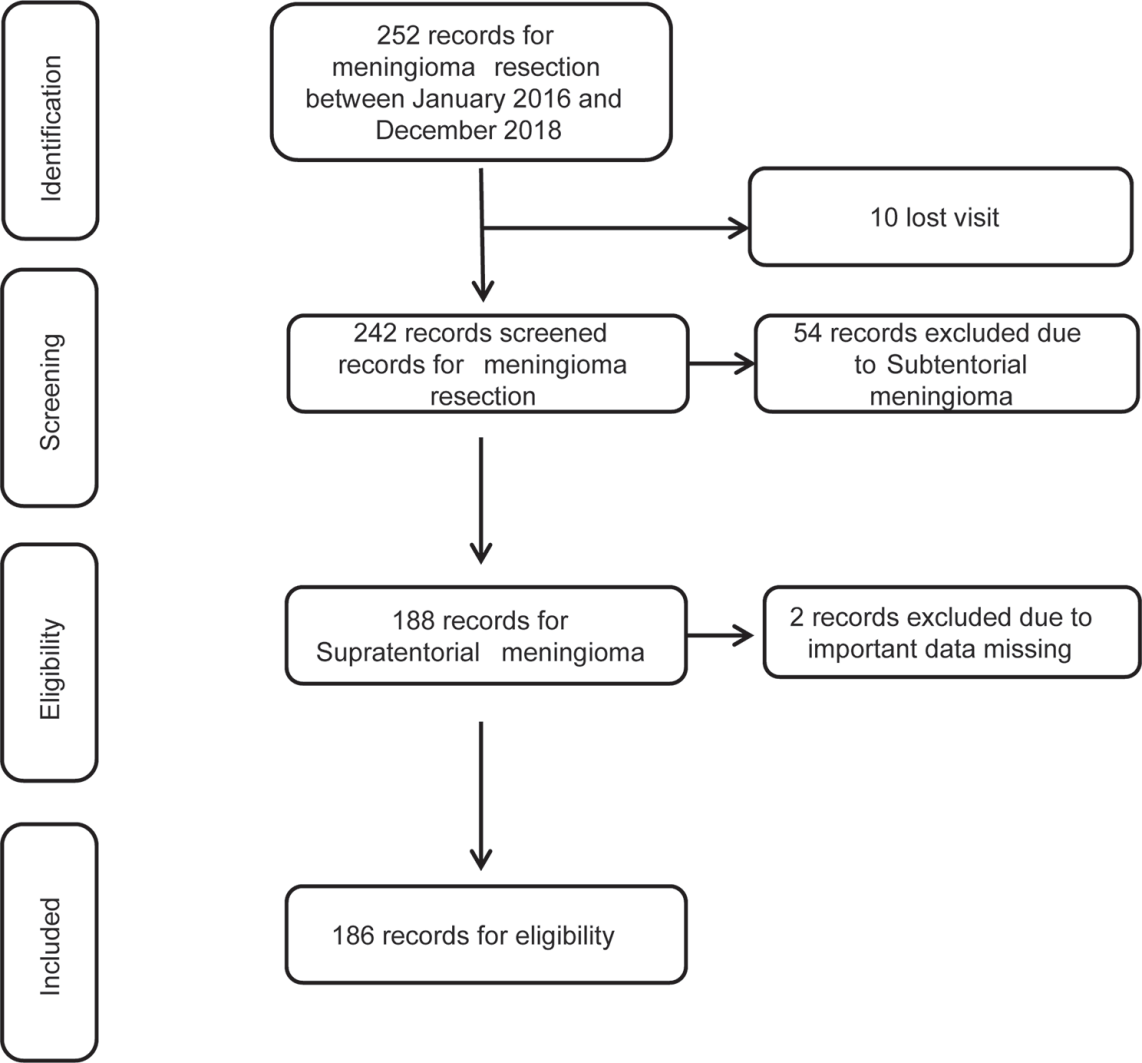
Flow chart of patients.

All patients came from the Department of Neurosurgery of the Affiliated Hospital of Guizhou Medical University, were diagnosed with meningioma, and underwent meningioma surgery between 2016 and 2018. All were older than 18 years of age. Of the 252 registered cases, 186 (73.8%) were deemed eligible, and 66 (26.2%) were excluded from the analysis. Specifically, 10 patients could not be followed-up, 54 patients were diagnosed with subtentorial meningioma, and two patients had loss of important data (**Figure 1**).

We collected the all the data related with the meningioma, including: age (years) at operation, sex, seizures before operation, the course of the disease (years), maximum cross-sectional area of the tumor (cm^2^), location of the tumor, multiple or single tumors, adjacent to the cortex, peritumoral brain edema, hydrocephalus, WHO classification, peritumoral adhesion, and use of AEDs in the perioperative period (20).

### Prophylactic AED Treatment During the Perioperative Period

Prophylactic AED treatment, in this case valproic acid, at 0.4 g added physiological saline (40 ml) to prevent seizures by continuous venous pump at 4 ml/h (3 days before surgical operation and 7 days after surgical operation). If the patients received valproic acid by oral administration before the operation, oral intake of valproic acid was ceased and switched to an intravenous pump. If the patients have taken other AEDs before operation, the patients would continue to use this drug.

### Data Grouping

Age (years) at surgery was categorized into two groups: ≥60-years-old and <60-years-old; course of disease (years) was divided three groups: <1 year, 1–5 years, and >5 years; maximum cross-sectional area of the tumor was categorized two groups: >15.0 cm^2^ and ≤15.0 cm^2^; location of the tumor was divided two groups: skull base location and non-skull base location; and WHO classification was divided into three groups: WHO grades I, II, and III according to the WHO classification of 2010 (12). Finally, according to whether AEDs were used or not and the number of AEDs, the patients were divided three groups: the no AEDs group, the prophylactic valproic acid treatment group, and the multiple AED treatment group (seizures before operation).

### Outcome of Postoperative Meningioma

On the 15th day after surgery, we recorded the patient’s seizures within 14 days. Fasting blood was also drawn for routine blood samples and liver and kidney function.

### Statistical Analysis

Continuous variables were expressed as mean ± standard deviation (normal distribution) or median (quartile; skewed distribution). Categorical variables were expressed in frequency or as a percentage. The chi-square tests (categorical variables) were used to determine any statistical differences between proportions of the groups. First, we performed a baseline characteristic of all patients to obtain an overview of the distribution of the data. A two-tailed chi-square test was then performed. P values less than 0.05 (two-sided) were considered statistically significant. Second, we performed univariate logistic regression analysis to assess the impacts of the determined variables on the occurrences of early postoperative seizures, including variables with odds ratios (ORs) with 95% confidence indexes (Cis). Third, multivariate logistic regression analyses were performed. All the meaningful factors were included. SPSS 24.0 for Windows (IBM, Armonk, New York, USA) was used for statistical analyses.

## Results

A total of 186 patients with meningioma were included in this study. All patients were categorized two groups according to seizure after operation. Detailed information is provided in **Table 1**. There were no statistically significant differences in age, sex, the course of the disease, maximum cross-sectional area of the tumor, seizures before surgery, location of the tumor, multiple tumors, adjacent to the cortex, peritumoral brain edema, WHO classification, or peritumoral adhesion between two groups (P >0.05). However, the results showed the patients with hydrocephalus, non-skull base location and were more prone to perioperative seizures (P <0.05). We also found that prophylactic AED treatment did not lower the rates of perioperative seizures (P <0.05).

**Table 1:** Baseline characteristics of participants.

Variable	Seizure after operation		P value
	Yes	No	
Age (years) at operation			0.526
≥60	10/54	44/54	
<60	30/132	102/132	
Gender			0.638
male	10/52	42/52	
female	30/134	104/134	
Seizure before operation			0.090
Yes	12/38	26/38	
No	28/148	120/148	
The course of disease(years)			0.983
<1	31/146	115/146	
1–5	6/27	21/27	
>5	3/13	10/13	
Maximum cross-sectional area			0.579
≤15	23/114	91/114	
>15	17/72	55/72	
Location of tumor			0.032
non-skull base location	34/142	108/142	
skull base location	6/44	36/44	
Multiple tumor			0.502
Yes	3/10	7/10	
No	37/176	139/176	
Adjacent to the cortex			0.150
Yes	27/107	80/107	
No	13/79	66/79	
Peritumoral brain edema			0.149
Yes	27/108	81/108	
No	13/78	65/78	
Hydrocephalus			0.003
yes	7/13	6/13	
No	33/173	140/173	
WHO classification			0.245
I level	35/171	136/171	
II/III level	5/15	10/15	
Peritumoral adhesion			0.774
Yes	27/122	95/122	
No	13/64	51/64	
AEDs			0.021
no	7/80	73/80	
Prophylactic treatment	24/68	44/68	
Treatment for seizure attack before operation	9/38	29/38	

### Study Population for a Univariate Logistic Regression Analysis

**Figure 2:**
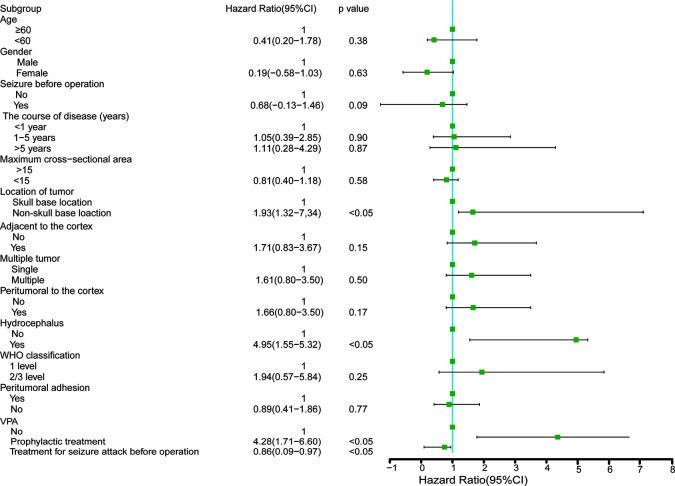
Study population for a univariate logistic regression analyses.

The results of univariate analysis are shown in **Figure 2** and showed that factors including age, sex, the course of the disease, maximum cross-sectional area of the tumor, seizures before surgery, location of the tumor, multiple tumors, adjacent to the cortex, peritumoral brain edema, WHO classification, and peritumoral adhesion were not associated with seizures after operation. Furthermore, we found prophylactic valproic acid treatment did not contribute to alleviation of seizures (P >0.05). Again, hydrocephalus and non-skull base location to the tumor likely contributed to perioperative seizure attack (P <0.05).

### Study Population for Multivariate Logistic Regression Analysis

The results of multivariate analysis are shown in **Table 2**. Age, sex, tumor location, hydrocephalus, and prophylactic valproic acid treatment were included. The results showed that there were no statistically significant differences in age, sex. Hydrocephalus (OR 4.87 P = 0.05) and non-skull base location (OR 1.88 P = 0.04) were significant risk factors for perioperative in-hospital seizures. We found incidence of epileptic seizure in the no prophylactic AEDs group were lower than that in the prophylactic AEDs group (OR 1.76 P = 0.04).

**Table 2:** Multivariate logistic regression analysis of study population.

	HR	CI 95%	P value
Age			
≥60	Reference		
<60	0.86	0.24–6.35	0.48
Gender			
Male	Reference		
Female	1.02	0.45–4.23	0.54
Hydrocephalus			
No	Reference		
Yes	4.87	1.34–13.56	0.05
Location of tumor			
skull base location	Reference		
non-skull base location	1.88	1.43–11.34	0.04
AEDs			
No	Reference		
Prophylactic treatment	1.76	1.52–9.56	0.04
Treatment for seizure attack before operation	1.21	0.37–8.15	0.43

### Study Population Excluding the Patients With Seizures Before Operation

We again performed analyses of baseline characteristics of patient data but excluded patients with seizures before operation. Detailed information is provided in **Table 3**. The results showed that there was no statistically significant differences in age, sex, the course of the disease, maximum cross-sectional area of the tumor, seizures before operation, location of the tumor, multiple tumors, adjacent to the cortex, peritumoral brain edema, WHO classification, or peritumoral adhesion (P >0.05). We found patients with hydrocephalus were more prone to occur perioperative seizures compared with patients without hydrocephalus (P <0.05). We confirmed that patients with non-skull base location to the tumor were more prone to perioperative seizures and that prophylactic AED treatment had no effect on the rate of seizures (P <0.05).

**Table 3:** Baseline characteristics of participants excluding the patients with seizure attack before operation.

Variable	Seizure after operation		P value
	Yes	No	
Age (years) at operation			0.600
≥60	7/43	36/43	
<60	21/105	84/105	
Gender			0.257
male	5/39	34/39	
female	23/109	86/109	
The course of disease(years)			0.028
<1 year	22/118	96/118	
1–5years	4/20	16/20	
>5 years	2/10	8/10	
Maximum cross-sectional area			0.384
≤15	15/90	75/90	
>15	13/58	45/58	
Location of tumor			0.045
non-skull base location	33/108	75/108	
skull base location	6/40	34/40	
Multiple tumor			0.504
Yes	2/7	5/7	
No	26/141	115/141	
Adjacent to the cortex			0.756
Yes	17/86	69/86	
No	11/62	51/62	
Peritumoral brain edema			0.645
yes	15/86	71/86	
no	10/62	52/62	
Hydrocephalus			0.002
yes	6/11	5/11	
No	22/137	115/137	
WHO classification			0.575
I level	25/136	111/136	
II/III level	3/12	9/12	
Peritumoral adhesion			0.440
yes	17/99	82/99	
no	11/49	38/49	
AEDs			0.001
no	7/80	73/80	
Prophylactic AED treatment	21/68	47/68	

### Study Population Excluding Patients With Seizures Before Operation for a Univariate Logistic Regression Analysis

**Figure 3:**
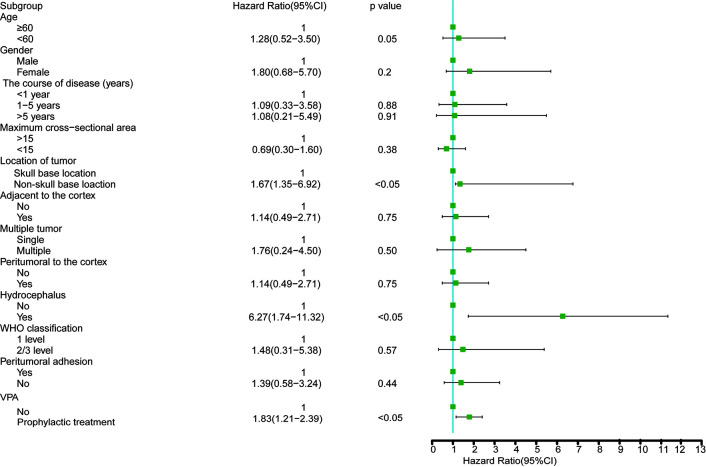
Study population excluding the patients with seizure attack before operation for a univariate logistic regression analyses.

The results of univariate analysis are shown in **Figure 3** and showed that age, sex, course of the disease, sectional area, tumor side, multiple tumors, location adjacent to the cortex, brain edema around the tumor, WHO classification and peripheral adhesion were not associated with perioperative seizures (P >0.05). We further confirmed that prophylactic valproic acid treatment did not reduce perioperative seizures (P <0.05). Hydrocephalus and non-skull base location to the tumor likely contributed to seizures (P <0.05).

### Study Population Excluding Patients With Seizures Before Operation for Multivariate Logistic Regression Analysis

The results of multivariate analysis are shown in **Table 4**. Age, sex, abundant blood supply, hydrocephalus and prophylactic valproic acid treatment were included in the analysis. Age, sex were not associated with perioperative seizures (P >0.05). Hydrocephalus and non-skull base location to the tumor likely contributed to seizures. We further confirmed that prophylactic valproic acid treatment did not reduce perioperative seizures (OR 1.84 P = 0.04).

**Table 4:** Multivariate logistic regression analysis of study population excluding the patients with seizure attack before operation.

	HR	CI95%	P value
Age			
≥60	Reference		
<60	1.32	0.52–5.16	0.48
Gender			
Male	Reference		
Female	1.78	0.67–6.78	0.65
Hydrocephalus			
No	Reference		
Yes	5.12	1.45–9.56	0.04
Location of tumor			
skull base location	Reference		
non-skull base location	2.34	1.23–6.95	0.03
AEDs			
No	Reference		
Prophylactic treatment	1.84	1.35–5.21	0.04

### Side Effect of Drug at 15 Days

Hepatorenal function was performed at 15 days post-surgery. There were three patients with mild increases of transaminase in liver function; all recovered after treatment.

## Discussion

Meningiomas, the most common type of brain tumor, are usually effectively treated surgically. However, perioperative seizures are a recurrent problem. Some surgeons choose to control epilepsy by prophylactic AEDs. In the neurosurgery department of our hospital, prophylactic AEDs have been given to patients with meningioma, although the efficacy of prophylactic AEDs in reducing the frequency of new-onset seizures in the perioperative period remains controversial (10). To further clarify if prophylactic AED treatment for patients with meningioma reduces the occurrence of seizures, we performed a retrospective study. The results showed patients with hydrocephalus and abundant blood supply were more prone to postoperative seizures. Importantly, we found that prophylactic AED treatment did not reduce the rate of postoperative seizures.

Intraoperative hemorrhage is more complicated for patients with abundant blood supply. Some of them with cerebral cortex contusion, even local hematoma formation, postoperative edema may be the main cause of seizure. As reported in the literature, postoperative complications can lead to seizures (21). Hydrocephalus is one of the causes of epilepsy. Previous studies have shown the stimulation of hydrocephalus to neurons may lead to neuronal discharge (22). Although the operation has relieved cortical compression, the brain tissue edema around the lesion and a large amount of exudation may cause seizure attack (23). Neuronal discharge will gradually decrease with the reduction of brain edema and absorption of exudation. Patients with non-skull base locations of meningioma seem to be more vulnerable to seizure attack. The main reason is more involved with the epileptogenic neocortical gray matter than those located at the skull base (21).

A report in 2011 analyzed the data of patients who underwent supratentorial meningioma resection from 1979 to 2010 (24). The authors found there were no significant differences between the incidence of early seizures and prophylactic AED therapy in patients undergoing supratentorial meningioma resection. Another study in the same year reported 180 patients with no preoperative history of seizures who underwent resection of a convexity meningioma. The patients received antiepileptic prophylaxis for 7 continuous days postsurgery. The rates of clinically evident seizures in the first 3–4 weeks after surgery were compared and indicated that routine use of prophylactic antiepileptics could prevent seizures in patients undergoing surgery for a convexity meningioma (25). Two studies in the same year reported different results.

In 2016, a meta-analysis of data collected from January 1980 to September 2014 was conducted and indicated that routine use of prophylactic anticonvulsants in patients without seizures was unnecessary (10). Islim et al. published the review in 2017 (14). The data were collected from between January 1990 and November 2016. The conclusion showed the prophylactic valproic acid treatment could reduce the rate of perioperative seizures. They performed a retrospective cohort study in 2018 (15), with the same conclusion. However, more studies showed the opposite conclusion (16–19). Routine use of prophylactic anticonvulsants in patients without seizures was unnecessary.

In our study, 186 cases were analyzed retrospectively. Patients received antiepileptic prophylaxis for 7 continuous days postsurgery. The results showed that incidence of perioperative seizures did not decrease. In order to exclude the effect of seizures before operation, we analyzed 148 patients with no preoperative history of seizures who underwent meningioma resection. The results showed there were no significant differences between the incidence of early seizures and prophylactic AED therapy. We think prophylactic use of AEDs had no effect on the rate of seizures for several reasons. First, meningiomas are relatively benign, mostly with intact borderlines. The operation was helpful to remove the tumor completely without damaging the surrounding brain tissue. Therefore, the possibility of seizures attack after operation was relatively low. The review written by Islim AI, et al. showed the results that early post-operative seizures occurred in 2.6% of patients (20 of 766) in the AED cohort. In the no-AED cohort of 377 patients, early post-operative seizures occurred in 2.7% of patients (10 of 377) (14). Second, there are many reasons leading to seizure attack after meningioma resection. Seizure attack could be reduced if the relevant factors were controlled. Third, sodium valproate is a common drug for controlling the seizure. However, more adverse effects were found.

Certainly, our study had some limitations. First, the sample size was not large enough to make multivariate logistic regression analyses for all the factors. Therefore, we made univariate logistic regression analyses, and screened for the meaningful factors. Then, we performed multivariate logistic regression analysis. Second, our study was a retrospective study, not a randomized controlled trial. It is difficult to balance all factors between the observation group and control group (for example, see **Table 1**). We should therefore perform univariate logistic regression analysis and multivariate logistic regression analysis to correct our data. Our study was a retrospective study, we did not collect more detailed data, such as brain infiltration, subdural hemorrhage, or any venous injury surgery. Third, we just confirmed that sodium valproate as prophylactic medication did not contribute to alleviation of perioperative seizures.

Taken together, we believe that prophylactic valproic acid treatment did not contribute to alleviation of perioperative seizures. We think randomized controlled trials should be a better way to study this problem.

## Data Availability Statement

The original contributions presented in the study are included in the article/supplementary material, further inquiries can be directed to the corresponding authors.

## Ethics Statement

The studies involving human participants were reviewed and approved by the Ethics Committee of Affiliated Hospital of Guizhou Medical University. The ethics committee waived the requirement of written informed consent for participation.

## Author Contributions

MY and Z-CX contributed to the drafting of the manuscript. Y-RC and M-YZ contributed to analysis and interpretation of the data. M-WW and LY contributed the collection of data. Z-HF and X-TM contributed to the conception and critical revision of the manuscript. All authors contributed to the article and approved the submitted version.

## Funding

This work was supported by National Natural Science Foundation of China (No. 81860248; No. 81960224); Science Foundation of China Association Against Epilepsy (No. 2018016) and Science and Technology Fund of Guizhou Health Commission (No. gzwjkj2020-2-005).

## Conflict of Interest

The authors declare that the research was conducted in the absence of any commercial or financial relationships that could be construed as a potential conflict of interest.
