# Corrigendum: Prophylactic AEDs Treatment for Patients With Supratentorial Meningioma Does Not Reduce the Rate of Perioperative Seizures: A Retrospective Single-Center Cohort Study

**DOI:** 10.3389/fonc.2020.638877

**Published:** 2021-01-18

**Authors:** Ming Yang, Yong-Ran Cheng, Meng-Yun Zhou, Ming-Wei Wang, Lan Ye, Zu-Cai Xu, Zhan-Hui Feng, Xun-Tai Ma

**Affiliations:** ^1^ Neurosurgical Department, Affiliated Hospital of Guizhou Medical University, Guiyang, China; ^2^ School of Public Health, Hangzhou Medical College, Hangzhou, China; ^3^ Department of Cardiology, Affiliated Hospital of Hangzhou Normal University, Hangzhou, China; ^4^ The Medical Function Laboratory of Experimental Teaching Center of Basic Medicine, Guizhou Medical University, Guiyang, China; ^5^ Neurological Department, Affiliated Hospital of Zunyi Medical University, Zunyi, China; ^6^ Neurological Department, Affiliated Hospital of Guizhou Medical University, Guiyang, China; ^7^ Neurological Department, The First Affiliated Hospital of Chengdu Medical College, Chengdu, China

**Keywords:** seizure, operation, prophylactic treatment, antiepileptic drugs, supratentorial meningioma, non-skull base location

In the published article, there was an error in the equal contribution labels. Instead of "Ming Yang^1^, Yong-Ran Cheng^2^, Meng-Yun Zhou^2†^, Ming-Wei Wang^3^, Lan Ye^4^, Zu-Cai Xu^5^, Zhan-Hui Feng^6^*^†^ and Xun-Tai Ma^7^*^†^", it should be "Ming Yang^1†^, Yong-Ran Cheng^2†^, Meng-Yun Zhou^2†^, Ming-Wei Wang^3^, Lan Ye^4^, Zu-Cai Xu^5^, Zhan-Hui Feng^6^*^‡^ and Xun-Tai Ma^7^*^‡^".

The authors apologize for this error and state that this does not change the scientific conclusions of the article in any way. The original article has been updated.

